# Heterotrimeric G-Protein Signalers and RGSs in *Aspergillus fumigatus*

**DOI:** 10.3390/pathogens9110902

**Published:** 2020-10-28

**Authors:** Hee-Soo Park, Min-Ju Kim, Jae-Hyuk Yu, Kwang-Soo Shin

**Affiliations:** 1 School of Food Science and Biotechnology, Kyungpook National University, Daegu 41566, Korea; 13mjkim@gmail.com; 2 Department of Integrative Biology, Kyungpook National University, Daegu 41566, Korea; 3 Department of Bacteriology, University of Wisconsin, Madison, WI 53706, USA; jyu1@wisc.edu; 4 Department of Systems Biotechnology, Konkuk University, Seoul 05029, Korea; 5 Department of Microbiology, Graduate School, Daejeon University, Daejeon 34520, Korea

**Keywords:** *Aspergillus fumigatus*, regulators of G-protein signaling, virulence, G-protein, G-protein-coupled receptors

## Abstract

The heterotrimeric G-protein (G-protein) signaling pathway is one of the most important signaling pathways that transmit external signals into the inside of the cell, triggering appropriate biological responses. The external signals are sensed by various G-protein-coupled receptors (GPCRs) and transmitted into G-proteins consisting of the α, β, and γ subunits. Regulators of G-protein signaling (RGSs) are the key controllers of G-protein signaling pathways. GPCRs, G-proteins, and RGSs are the primary upstream components of the G-protein signaling pathway, and they are highly conserved in most filamentous fungi, playing diverse roles in biological processes. Recent studies characterized the G-protein signaling components in the opportunistic pathogenic fungus *Aspergillus fumigatus*. In this review, we have summarized the characteristics and functions of GPCRs, G-proteins, and RGSs, and their regulatory roles in governing fungal growth, asexual development, germination, stress tolerance, and virulence in *A. fumigatus*.

## 1. Introduction

*Aspergillus fumigatus*, a saprophytic fungus, is found in various ecological niches [1,2,3]. This fungus is also a key opportunistic human pathogenic fungus that causes invasive aspergillosis primarily in immunocompromised patients and other human diseases, including allergic bronchopulmonary aspergillosis, fungal asthma, and chronic pulmonary disease [4,5,6,7]. Recently, there have been reports of increased risks and mortality due to the coinfection of COVID-19 and invasive pulmonary aspergillosis, which is also called COVID-19-associated pulmonary aspergillosis [8,9]. To survive in the host system or harsh environmental conditions, this fungus senses different signals and transmits them into the cell [10,11]. For this, various signal transduction pathways are involved in appropriate biological processes [10]. In addition, the signal transduction pathways regulate the production of virulence factors, including gliotoxin and melanin, thereby affecting the pathogenesis of *A. fumigatus* [4,12]. Therefore, it is necessary to understand the signal transduction pathways in *A. fumigatus*.

The heterotrimeric guanine nucleotide-binding protein (G-protein) signaling pathway is an important signal transduction pathway that has multiple functions in most eukaryotic systems [13,14]. G-proteins are composed of three subunits, α (Gα), β (Gβ), and γ (Gγ), which are activated by G-protein-coupled receptors (GPCRs), and transmit signals into the cell for an appropriate biological response [15,16]. In the presence of a ligand, GPCRs can function as guanine nucleotide exchange factors, accelerating the exchange from Gα-guanosine diphosphate (GDP) to Gα-Guanosine triphosphate (GTP) and the dissociation of the Gα subunit from the heterotrimeric G-protein Gαβγ complex [17,18]. Gα-GTP and Gβγ heterocomplexes can transmit signals leading to downstream effectors. The activated G-protein signaling is turned off when Gα-GTP is hydrolyzed to Gα-GDP by GTPase-accelerating proteins (GAPs) [19]. Regulator of G-protein signaling (RGS) proteins are one of the GAPs that induce the intrinsic GTPase activity of Gα-GTP, thereby attenuating G-protein signaling [20,21].

GPCRs, heterotrimeric G-proteins, and RGS proteins are the key components of G-protein signaling and highly conserved in most filamentous fungi [22,23]. In particular, these G-protein signaling components have multiple functions in fungal growth, asexual development, secondary metabolism, and stress responses in filamentous fungi [22]. In the opportunistic fungal pathogen *A. fumigatus*, 15 classical GPCRs, 3 Gα subunits, 1 Gβ subunit, 1 Gγ subunit, and 6 RGS proteins have been identified [23,24,25]. For decades, researchers have studied the functions of G-protein signaling components and revealed that the G-protein signaling pathway has diverse roles in the growth, development, stress response, and virulence in *A. fumigatus*. This review provides knowledge about the functions of G-protein subunits and RGS proteins in *A. fumigatus*.

## 2. GPCRs

GPCRs sense various environmental signals and transmit them for appropriate biological responses via the heterotrimeric G-protein complex [26]. In the *A. fumigatus* genome, 15 genes encode putative classical GPCRs [23]. Among them, only five GPCRs have been characterized. GprC and GprD are similar to Gpr1, the carbon-sensing receptor in *Saccharomyces cerevisiae* that activates the cyclic AMP (cAMP)–protein kinase A (PKA) pathway [27,28]. Gehrke et al. characterized the roles of GprC and GprD and demonstrated that GprC and GprD are required for hyphal growth, germination, and stress response against thermal stress and reactive oxygen species [29]. The Δ*gprC* or Δ*gprD* mutant strains are attenuated for virulence in the murine low-dose model for invasive aspergillosis. Transcriptomic analyses found that GprC and GprD are involved in the mRNA expression of genes associated with primary and secondary metabolism. GprK contains seven transmembrane domains with an RGS domain [30]. The detailed functions of GprK are described below. Recently, two GPCRs, GprM and GprJ, have been characterized. GprM and GprJ are essential for normal melanin production, cell wall integrity, and virulence. RNA-sequencing and mass spectrometry analyses demonstrated that the two GPCRs regulate the mRNA expression of secondary metabolite gene clusters, thereby affecting the production of various secondary metabolites [31]. The functions of the five GPCRs have been studied, but further studies on the other GPCRs are still needed.

## 3. Heterotrimeric G-Protein Complex

**Figure 1:**
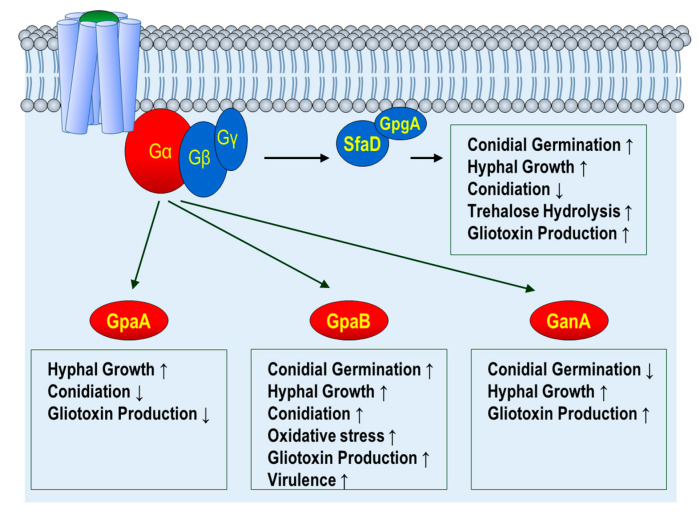
Functions of G-protein subunits in *A. fumigatus*. Gα; G alpha subunit, Gβ, G beta subunit, Gγ; G gamma subunit. GpaA, GpaB, and GanA are Gα subunits. SfaD is a Gβ subunit. GpgA is a Gγ subunit Upwards arrow(↑) indicates activation/induction. Downwards arrow(↑) indicates repression/inhibition.

The heterotrimeric G-protein complex consists of three subunits, Gα, Gβ, and Gγ [22]. In *A. fumigatus*, three Gα subunits, one Gβ subunit, and one Gγ subunit have been identified, which is similar to other filamentous fungi. Their functions have been characterized [32,33] (Figure 1).

GpaA is the *A. nidulans* FadA homolog. The absence of *gpaA* causes increased conidiation and reduced gliotoxin production [33]. The conidiation reduction in the Δ*gpaA* mutation is similar to the dominant-interfering (GpaA^G203R^) mutation, a G-protein signaling-blocking mutation, but it is the opposite of the dominant active mutation (GpaA^Q204L^), reducing the intrinsic GTPase activity of GpaA and resulting in uncontrolled GpaA activity [24,33]. In addition, the dominant-activating mutation of GpaA results in increased hyphal growth [24]. Genetic analysis demonstrates that the reduction of conidiation in the dominant-interfering GpaA mutation is suppressed by the loss-of-function *flbA*, suggesting that GpaA is the primary target of FlbA, which is an RGS protein (see below) [24]. These results indicate that GpaA-mediated signaling induces hyphal growth but inhibits conidiation in *A. fumigatus*.

GpaB belongs to Group II Gα proteins involved in different biological processes in fungi. Liebmann et al. found that the Δ*gpaB* mutant strain is attenuated or almost avirulent in the murine inhalation model [34]. In addition, the Δ*gpaB* strain produces fewer conidia, suggesting that GpaB is required for proper conidiation. A mechanistic study found that GpaB regulates growth and asexual development via the cAMP–PKA signal transduction pathway [34]. Further phenotypic analyses have found that *gpaB* deletion results in decreased gliotoxin production and conidial germination [33]. According to a recent study on the third Gα protein, GanA is involved in germination and gliotoxin production [33].

In the eukaryotic system, Gβ and Gγ subunits are highly conserved. They form the Gβγ heterodimeric complex, acting as one functional unit. SfaD and GpgA are the Gβ and Gγ subunits, respectively, in *A. fumigatus*. The phenotypes of the Δ*sfaD* and Δ*gpgA* strains are almost same, and these two mutations have more severe phenotypes than any Gα mutant strain [32]. Functional studies have found that the Δ*sfaD* and Δ*gpgA* strains exhibit delayed trehalose hydrolysis, conidial germination, hyphal growth, and gliotoxin biosynthesis but induce conidiophore formation and the expression of *brlA*, which is a key activator for conidiation in *A. fumigatus* [32]. Importantly, the functions of Gβγ subunits in germination and vegetative growth are highly conserved in other *Aspergillus* species.

## 4. RGS

**Figure 2:**
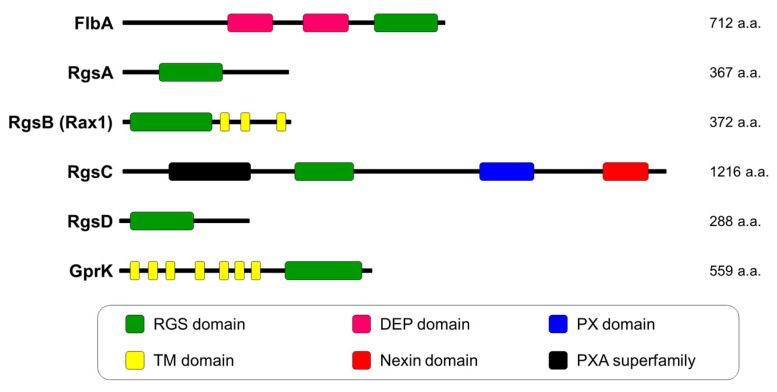
Domain architecture of regulators of G-protein signaling (RGS) proteins in *A. fumigatus*. Abbreviations are regulators of G-protein signaling domain, RGS domain; Dishevelled, Egl-10, and Pleckstrin domain, DEP domain; PhoX domain, PX domain; transmembrane domain, TM domain; PhoX-associated superfamily, PXA superfamily.

RGS proteins are the key factors that interact with active Gα subunits to accelerate the rate of GTP hydrolysis [21]. In fungi, RGS proteins have multiple functions in different biological processes [35,36]. In the pathogenic fungus *A. fumigatus*, six proteins, including FlbA, RgsA, RgsB (Rax1), RgsC, RgsD, and GprK, contain RGS domains. Their functions have been identified (Figure 2). These six RGS proteins have diverse functions in fundamental biological processes (Table 1).

### 4.1. FlbA

FlbA was the first characterized RGS protein in the model fungus A. nidulans [44]. The flbA gene is one of the fluffy genes (fluG, flbA-E) that encode a protein containing two DEP (Dishevelled, Egl-10, and Pleckstrin) domains in the middle region and one RGS domain in the C-terminal region. The loss-of-function flbA mutants exhibited the fluffy phenotype with cotton-like colonies, which are characterized by undifferentiated masses of vegetative hyphae [45], and a reduction in the mRNA level of brlA, which is a key initiator for conidiation in A. nidulans [44]. In addition, flbA overexpression causes the induction of brlA expression and premature asexual development under normally repressive conditions, i.e., submerged culture [46]. Results of genetic analyses found that FadA (Gα) is a target Gα subunit for FlbA. FlbA controls vegetative growth, development, and secondary metabolism via the FadA-mediated signaling pathway in A. nidulans [35,44,46]. The function of FlbA and Gα-RGS signaling components is conserved in A. nidulans and A. fumigatus. Mah and Yu found that the ΔflbA mutants exhibit decreased conidiation in A. fumigatus [24]. In addition, decreased conidiation of the GpaA (homologue of A. nidulans FadA) dominant-interfering mutation is restored by loss-of-function flbA, suggesting that FlbA attenuates vegetative growth via the inactivation of GpaA in A. fumigatus. In addition to its function in vegetative growth, other functions of FlbA have been studied via comparative proteomic analyses. flbA deletion affects the secretion of GliT and intracellular superoxide dismutase (SOD) activity, implying that FlbA is involved in the detoxification of exogenous gliotoxin and reactive oxygen species in A. fumigatus [37]. FlbA is also involved in detoxification by regulating catalase expression and activity [37]. Overall, FlbA has crucial functions in vegetative growth, asexual development, and detoxification in *A. fumigatus*.

### 4.2. RgsA

The *rgsA* gene encodes a protein that contains a typical RGS domain. RgsA has diverse roles in *A. fumigatus* [37]. First, *rgsA* deletion causes induced conidiophore production and increased mRNA levels of the asexual developmental regulatory genes *brlA*, *abaA*, and *wetA*, suggesting that RgsA attenuates asexual development. Second, Δ*rgsA* mutants exhibit increased germination rates and hyphal growth. Third, the absence of *rgsA* leads to increased tolerance against oxidative stress-inducing compounds and induced levels of genes related to stress response. These phenotypes of the Δ*rgsA* strain in *A. fumigatus* are quite similar to those of the Δ*rgsA* strain in *A. nidulans* [47], indicating that the function of RgsA can be conserved in *Aspergillus* species. The target Gα protein (GanB) of RgsA has been identified in *A. nidulans*. RgsA attenuates fungal growth, germination, conidiation, and oxidative stress response via the GanB-mediated signaling pathway. However, it has not been identified in *A. fumigatus*. One possible target Gα protein for RgsA might be GpaB, because of various phenotypes, including gliotoxin production, growth, and conidiation, of the Δ*gpaB* strain exhibit the opposite phenotypes of the Δ*rgsA* strain [33,39]. However, this should be verified through further experiments, such as protein–protein interaction or genetic analysis. The downstream pathway of RgsA-mediated regulation is not clear. However, it has been proposed that RgsA negatively regulates the PKA signaling pathway and oxidative stress response pathway, thereby regulating fungal growth, oxidative stress tolerance, and conidiation [39]. In addition, RgsA is required for gliotoxin production, virulence, and carbohydrate metabolism in *A. fumigatus*. The amount of gliotoxin and mRNA levels of genes in the gliotoxin gene cluster increases in the Δ*rgsA* strain. In the immunocompromised mouse model, *rgsA* deletion causes an increased mortality rate and pulmonary fungal burden. The transcriptomic analysis found that the absence of *rgsA* affects the mRNA expression of genes encoding carbohydrate metabolism-related proteins and cellulolytic enzymes, including cellobiohydrolase, endo-1,4-β-glucanase, and β-glucosidase. In addition, deletion of *rgsA* results in decreased intracellular and extracellular endoglucanase activities, suggesting that RgsA plays a crucial role in endoglucanase activity.

### 4.3. RgsB (Rax1)

RgsB (or Rax1) is an ortholog of *S. cerevisiae* Rax1, which is required for the bipolar budding pattern [48]. RgsB has multiple functions in growth, development, and conidial tolerance. *rgsB* deletion resulted in restricted radial growth and reduced conidial production, suggesting that RgsB is a positive regulator for vegetative growth and conidiation [40]. RgsB is also required for conidial tolerance against oxidative stress. Δ*rgsB* conidia have more trehalose contents than wild-type (WT) conidia. They contain a thicker electron-dense melanized layer compared to WT, which appear to have a vital role in conidial tolerance against H_2_O_2_ [40]. RgsB is also essential for endoplasmic reticulum stress and exogenous gliotoxin response. However, RgsB does not affect virulence in the murine model [41].

### 4.4. RgsC

RgsC is the longest protein among the RGS proteins and has PhoX-associated (PXA), RGS, PhoX homology (PX), and nexin domains. PX domain acts as a phosphoinositides-binding motif, and PXA, PX, and nexin domains are involved in protein sorting and membrane traffic in the endosomal system [49]. Transcriptomic and phenotypic analyses of the Δ*rgsC* mutant indicate that RgsC is associated with conidiation, germination, stress response, and toxin production [42]. *rgsC* deletion results in an accelerated conidial germination rate, decreased conidia production, and increased oxidative stress sensitivity. In the *Galleria mellonella* infection model, the Δ*rgsC* strain is attenuated for virulence. Gliotoxin production and the mRNA expression of genes in gliotoxin gene clusters are decreased in the Δ*rgsC* strain compared to WT. Collectively, these indicate that the RgsC-mediated signaling pathway regulates proper asexual development, gliotoxin biosynthesis, fungal virulence, and stress response in *A. fumigatus*.

### 4.5. RgsD

Similar to RgsA, RgsD contains one RGS domain in the N-terminal region. A protein interaction study using a yeast two-hybrid assay indicated that RgsD mainly interacted with GpaB, suggesting that RgsD might regulate the GpaB-mediated signaling pathway [43]. In the downstream signaling pathway, RgsD negatively regulates the cAMP-dependent PKA signaling pathway. Phenotypic and transcriptomic analyses demonstrated that RgsD negatively regulates conidiation, stress response, melanin biosynthesis, and gliotoxin production. In the *G. mellonella* infection model, *rgsD* deletion results in enhanced virulence. These results may be due to the loss-of-function *rgsD* that causes the enhanced production of two virulence factors, such as melanin and gliotoxin. These results propose that RgsD attenuates the GpaB signaling pathway, which then affects cAMP–PKA signal transduction. RgsD modulates asexual development, melanin biosynthesis, stress response, and virulence in *A. fumigatus*.

### 4.6. GprK

GprK is a hybrid protein that contains both seven-transmembrane and regulator of RGS domains. GprK is a unique protein. GprK orthologs can be found in Ascomycetes but not in Basidiomycetes [22]. Functional study results for *A. fumigatus* demonstrated that GprK acts as a multifunctional regulator for development, oxidative stress response, gliotoxin production, and germination [30]. The absence of *gprK* results in impaired conidia production and reduced transcript expression of conidiation-related genes, such as *brlA*, *abaA*, and *wetA*. The Δ*gprK* strain is more sensitive to oxidative stress than WT. In addition, the mRNA expression of *sakA* and *atfA* and the activities of CatA, SOD1, and SOD2 are reduced in the Δ*gprK* strain, proposing that GprK governs the oxidative stress response through the AtfA-mediated signaling pathway [30].

## 5. Other Components

RicA is an ortholog of Ric-8 in animals or yeasts, which stimulates the dissociation of monomeric Gα-GDP but not the guanine nucleotide exchange activity of the Gα subunit [50,51]. The functions of RicA are conserved in *Aspergillus* species and crucial for growth and development in *A. fumigatus* [52]. The phenotypes of Δ*ricA* exhibit delayed and/or reduced conidial germination, vegetative proliferation, conidiophore formation, and proper hyphal cell death in liquid submerged culture. The transcript expression of key asexual developmental regulators, such as *brlA*, *abaA*, and *wetA*, is decreased in *A. fumigatus* Δ*ricA* mutants. These results suggest that RicA has a vital function in asexual development and growth. Although the mechanistic research was not conducted in *A. fumigatus*, the molecular mechanism of RicA was carried out in the model organism, *A. nidulans*. This study found that RicA can interact with GanB, which is a Gα subunit. The RicA-mediated signaling is primarily through the GanB–PkaA pathway in *A. nidulans*.

CpcB (cross-pathway control B) is a Gβ-like protein, which is the ortholog of *Cryptococcus neoformans* Gib2 and mammalian RACK1 ortholog. *cpcB* deletion affects hyphal growth, conidiophore morphology, and gliotoxin production in *A. fumigatus* [53,54]. In addition, the deletion of *cpcB* attenuates fungal virulence in an immunosuppressed mice model. The results of complementary experiments indicate that the function of CpcB is conserved in *Aspergillus*, but the function of CpcB is partially restored by the yeast CpcB ortholog [54]. Genetic analysis also indicates that CpcB has a distinct role compared to the subunits of the G-protein complex. Structurally, CpcB contains the seven WD-40 repeat motif required for the normal function of CpcB [55]. The phenotypes of several truncated *cpcB* mutant strains exhibit abnormal hyphal growth, conidiation, antifungal drug susceptibility, and virulence in *A. fumigatus*. Overall, these results support the idea that CpcB has a crucial function in the normal fungal growth, development, and pathogenesis in *A. fumigatus*.

## 6. Conclusions

In filamentous fungi, the G-protein signaling pathway is crucial for regulating different biological processes. The pathway senses external environmental signals and coordinates fungal metabolism, development, and stress responses. In addition, the components of the G-protein signaling pathway are involved in the pathogenesis or production of virulence factors, such as melanin and gliotoxin, suggesting that these components are targets for antifungal development. Although the function of each component was characterized, the detailed signaling cascades of the G-protein signaling still need further research. In addition, further research is also needed on the possibility of RGS as a target for antifungal agents. Therefore, future studies should provide insights into the detailed molecular mechanism underlying cell growth, morphogenesis, metabolism, and pathogenesis in human pathogenic fungi.

## Figures and Tables

**Table 1:** Functions of RGS proteins in *A. fumigatus*.

Name	Potential Gα Target	Potential Downstream Pathway	Function	References
FlbA	GpaA	PKA-mediated pathway	Hyphal growth, conidiation, detoxification	[24,37,38]
RgsA	—	PKA-mediated pathway AtfA-mediated pathway	Hyphal growth, conidiation, conidial germination, oxidative stress response, gliotoxin production, virulence, carbohydrate metabolism	[39]
RgsB ^a^	—	—	Hyphal growth, conidiation, melanin production, stress response	[40,41]
RgsC	—	PKA-mediated pathway	Conidiation, conidial germination, stress response, gliotoxin production	[42]
RgsD	GpaB	PKA-mediated pathway	Conidiation, stress response, melanin production, gliotoxin production, virulence	[43]
GprK	—	PKA-mediated pathway AtfA-mediated pathway	Conidiation, oxidative stress response, gliotoxin production, conidial germination	[30]

^a^ RgsB is also called Rax1.
